# Epidemiology, evolution and transmission of human metapneumovirus in Guangzhou China, 2013–2017

**DOI:** 10.1038/s41598-019-50340-8

**Published:** 2019-10-01

**Authors:** Lina Yi, Lirong Zou, Jingju Peng, Jianxiang Yu, Yingchao Song, Lijun Liang, Qianfang Guo, Min Kang, Changwen Ke, Tie Song, Jing Lu, Jie Wu

**Affiliations:** 1 Guangdong Provincial Center for Disease Control and Prevention, No. 160, Qunxian Road, Panyu District, Guangzhou, China; 2 Guangdong Provincial Institute of Public Health, No. 160, Qunxian Road, Panyu District, Guangzhou, China; 3 Southern Medical University, No. 1838, Shatai Road, Baiyun District, Guangzhou, People’s Republic of China

**Keywords:** Molecular evolution, Viral genetics

## Abstract

Human metapneumovirus (hMPV), first identified in 2001, is a major viral respiratory pathogen that worldwide reported. Fundamental questions concerning the dynamics of viral evolution and transmission at both regional and global scales remain unanswered. In this study, we obtained 32 G gene and 51 F gene sequences of hMPV in Guangzhou, China in 2013–2017. Temporal and spatial phylogenetic analyses were undertaken by incorporating publicly available hMPV G gene (978) and F gene (767) sequences. The phylogenetic results show different global distribution patterns of hMPV before 1990, 1990–2005, and 2006–2017. A sharply increasing hMPV positive rate (11%) was detected in Guangzhou 2017, mainly caused by the B1 lineage of hMPV. A close phylogenetic relation was observed between hMPV strains from China and Japan, suggesting frequent hMPV transmissions between these regions. These results provide new insights into hMPV evolution, transmission, and spatial distribution and highlight Asia as a new epicenter for viral transmission and novel variant seeding after the year 2005. Conducting molecular surveillance of hMPV in Asian countries is critical for understanding the global circulation of hMPV and future vaccine design.

## Introduction

Human metapneumovirus (hMPV), first identified in 2001, is a major viral respiratory pathogen that worldwide reported^1^. It is estimated that 4–16% of acute respiratory tract infections are caused by HMPV^2,3^. Retrospective serologic studies demonstrated 100% seroprevalence of hMPV antibodies among children ≤5 years of age^4^. Moreover, reinfection can occur throughout life^5,6^, further contributing to the prevalence of hMPV. Clinical features of HMPV infection are similar to those associated with human respiratory syncytial virus infection, ranging from mild respiratory illnesses to severe bronchiolitis and pneumonia^4,7^. Children under five years, aged adults, and patients with underlying diseases are more likely to develop severe diseases^8^.

HMPV is a single stranded, negative sense and enveloped RNA virus. The viral RNA is approximately 13 kb in length, containing eight genes coding for nine proteins^9^. The fusion (F) and attachment (G) proteins are two major hMPV surface glycoproteins that have played important roles in viral replication and host immune response^10^ and have been largely used to study hMPV genetic variation^11–13^. Based on the genetic variability of the F and G genes, hMPV is classified into two main genetic groups, A and B, which are further divided into five lineages known as A1, A2a, A2b, B1, and B2^14,15^. There may no direct association between the genotypes and the clinical courses^16^. However, antigenic variance is observed among different genetic lineages of hMPV in animal models and human infection cases^10,17^.

The genetic evolution and transmission of hMPV are critical to epidemic control but have not been thoroughly studied as yet. Recently, infections of hMPV were increasingly reported in Asian countries^6,18–24^, with novel variants of hMPV emerging^23,24^, highlighting the risks of hMPV epidemics in these regions. Here, we conducted a five-year study on hMPV in Guangzhou, the most populous city in China, from January 2013 to December 2017. Through phylogeographic analyses, the spatial and evolution dynamics of hMPV were inferred by integrating the publicly available data and the newly generated sequences in this study.

## Materials and Methods

### Ethics statement

This study was approved by the Ethics Review Committee of the Guangdong Provincial Center for Disease Control and Prevention. We confirm that all methods were performed in accordance with the relevant guidelines and regulations. Respiratory samples were collected for public health purposes. All patients or their guardian(s) were informed about the study before providing written consent, and data were anonymized for analysis.

### Clinical sample collection

Guangzhou in southern China is the country’s largest city with a population of over 13 million in 2016. Observation was conducted in a sentinel hospital in Guangzhou through a Guangdong provincial respiratory surveillance system as previously described^25,26^. Patients suspected of having acute respiratory tract infections were enrolled according to these criteria: acute fever (*T* ≥ 38 °C) and/or abnormal leukocyte count with any one respiratory symptom (such as sore throat, cough, expectoration, or dyspnoea/tachypnoea). A total of 1,460 patients were enrolled from January 2013 to December 2017. Nasopharyngeal swabs (NPSs) were collected within 24 h after admission. Specimens were stored in 3 mL viral transport medium at −70 until analysis.

### Detection of human metapneumovirus

Total viral nucleic acids were extracted from 140ul specimen and eluted with 60ul water by using a QIAamp Viral RNA Mini kit (Qiagen, Valencia, CA, USA) according to the manufacturer’s instructions. hMPV infection was detected by using real-time RT-PCR with a QIAGEN OneStep RT-PCR Kit. The hMPV RT-PCR primer set was used as previously described^27^: hMPV-F: 5′-CGTCAGCTTCAGTCAATTCAACAGA-3′, hMPV-R: 5’-ATTARGTCCAADGATATTGCTGGTGTT-3’ and hMPV-Probe: FAM-CTGCATTGTCTGAAAAYTGCCGCACAACATT-BHQ1. The thermocycling protocol was reverse transcription at 45 °C for 10 min, followed by 10 min at 95 °C, 40 cycles of 95 °C 15 s, and 55 °C 60 s.

### PCR amplification of the full-length F and G protein genes

For hMPV positive samples, the viral RNA was reverse transcribed into cDNA using a SuperScript™ IV First-Strand Synthesis System (Invitrogen, USA). Then the full length of F and G genes was amplified using traditional nested PCR with primers newly designed in this study. Primers used for G gene amplification were: hMPV-G-F: 5′-GAGAACATTCGRRCRATWGAYATG -3′, hMPV-G-R1:5′-AGATARACATTRACAGTRGAYTCA-3′, and hMPV-G-R2:5′-C AAYAACAGGGTTTTCYAYAGCA-3′. The primer sets for F gene were: outer primers set hMPV-F-F1-5′-CAATGCAGGTATAACACCAGCAATATC-3′ and hMPV -F-R1 5′-GCAACAATTGAACTGATCTTCAGGAAAC-3′, and inner primers set hMPV-F-F2-5′-ACATGCCAACATCTGCAGGACAAATAAAAC-3′ and hMPV-F- R2 5′- ACATGCTGTTCACCTTCAACTTTGC-3′. The PCR products were purified and sequenced using an ABI3730xl DNA Analyzer at IGE Biotech Co., Ltd. (Guangzhou, China). In total, 51 and 32 samples (out of the total 160 RT-PCR positive samples) F and G genes were sent for sequencing, respectively. Sequences generated in this study have been submitted to GenBank (accession numbers MK450165-MK450246).

### Sequence alignment and maximum-likelihood phylogenetic analysis

The hMPV sequences generated in this study were combined with all publicly available hMPV G and F genes sequences with known sampling dates and locations in GenBank. Partial sequences (<80%) of the G and F genes were excluded, and identical sequences collected in the same sampling location on the same date were removed to improve computation time. In total, 978 G gene sequences (568 genogroup-A and 410 genogroup-B) and 767 F gene sequences (365 genogroup-A and 402 genogroup-B) were included in the phylogenetic analysis (Table S1) with sequences generated in this study. Multiple sequence alignment was performed using ClustalW, and alignments were minimally edited by hand using Aliview^28^. Maximum-likelihood (ML) trees were estimated for G and F genes in RaxML^29^. Temporal accumulation of genetic divergence was assessed from maximum-likelihood, mid-point-rooted phylogenies using the linear regression approach implemented in TempEst (formerly Path-O-Gen)^30^.

### Phylogeographic analysis

Compared to the F gene, more hMPV G gene sequences from different regions were available in the public database. Therefore, we performed the phylogeographic analysis on genogroup-A and genogroup-B G gene sequences separately to provide an outline of hMPV distribution and transmission. As previously described^25,31,32^, Bayesian Markov chain Monte Carlo (MCMC) phylogenetic inference was performed using BEAST under a SRD06 nucleotide substitution model and a GMRF Bayesian skyride coalescent model^33^. Preliminary analysis indicated high values for the coefficient of variation parameter of the molecular clock model, therefore an uncorrelated lognormal (UCLD) relaxed clock model was used in the final analysis to accommodate variation in substitution rates among branches^34^. Three independent MCMC runs of 1 × 10^8^ steps were computed and 10–20% burn-in was discarded from each, resulting in a total of 2.0 × 10^8^ total steps. Model parameters and trees were sampled every 10,000 MCMC steps. Convergence and behavior of MCMC chains was inspected using Tracer v1.6 (http://tree.bio.ed.ac.uk). A subset of 1,000 trees was randomly drawn from the combined posterior distribution of trees and used as an empirical distribution for subsequent phylogeographic analysis^35^.

## Results

### Epidemiology of hMPV in southern china

**Figure 1:**
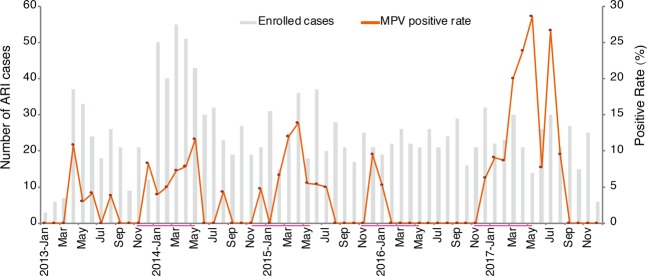
Month distribution of acute respiratory infection cases and hMPV positive rates in Guangzhou, 2013–2017. Duration from November to the next May is highlight to show the seasonality of hMPV epidemic.

A total of 1,460 ARI patients (927 males and 533 females) were enrolled in Guangzhou from January 2013 to December 2017 (Fig. 1). HMPV was detected in 76 samples (5.2%), including 50 males and 26 females (1.92:1). No significant gender difference was observed (p = 0.669). The age of enrolled patients ranged from 1 month to 88 years, with the majority (82.7%) being children no more than 5 years (median age: 11 months, IQR, 0.3–3 years). HMPV positive cases were aged from 1 month to 81 years (median age: 1 year, IQR, 0.4–2.5 years). Half (52.6%) of infected individuals were children no more than 1 year. The distribution of HMPV prevalence among age groups was significantly different (P = 0.016), and children aged 1 to 2 years had the highest prevalence (9.1%, 15/164). The clinical presentations of hMPV infected cases included fever (69, 90.8%), cough (69, 90.8%), sore throat (38, 50.0%), expectoration (16, 90.8%), shortness of breath (10, 13.2%) and nasal discharge (6, 7.9%). The seasonal distribution showed that hMPV circulated predominantly in the period from December of the first year to May of the next (Fig. 1). In the years 2013–2015, the hMPV detection rate was approximately even, ranging from 3.7% to 5.1%, similar with the rate detected in central and eastern China. An apparent low frequency of circulation of hMPV in the year 2016 was observed, with a low positive rate of 1.1%. However, in the year 2017, hMPV showed a remarkable increase, viruses being detected in 31 ARI cases (11.9%).

### Genetic divergence and lineage classification of hMPV

**Figure 2:**
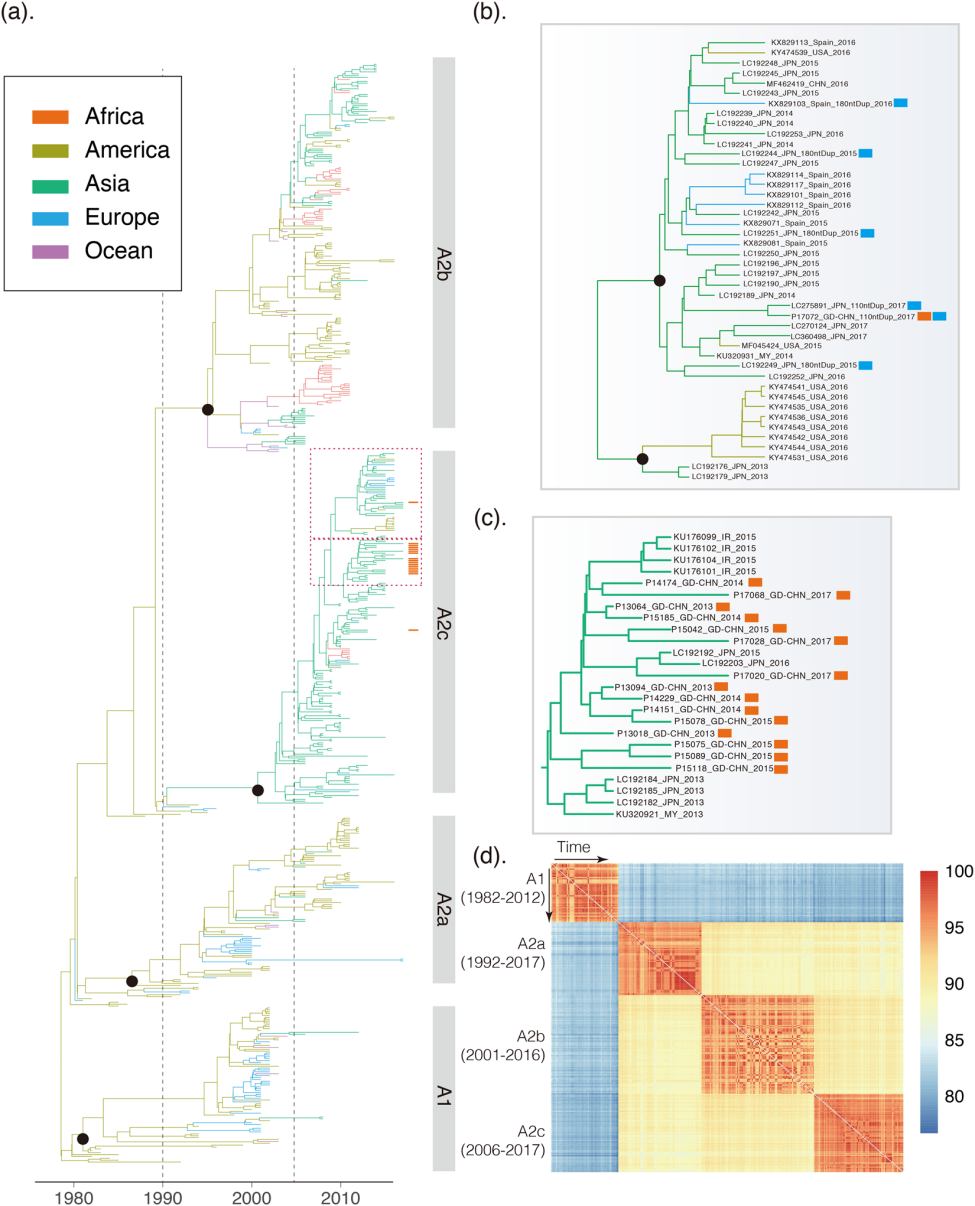
Genetic evolution and spatial spread of the genogroup A hMPV lineages. (**a**) Bayesian Maximum Clade Credibility (MCC) tree of G gene from genogroup A hMPV viruses. sequences reported in this study from Guangzhou are highlighted by orange bars. Branch colours represent the most probable ancestral locations of each branch. Four major lineages of hMPV are observed, denoted A1, A2a, A2b, and A2c. Black circles indicate posterior support >0.95 at the root node of each lineage. The clusters containing major circulating strains in Guangzhou are marked with box. These clusters are enlarged and names of strains are included in (**b**,**c**). (**b**) The cluster of hMPV including the 180-nt duplication novel variant (marked with light blue bars) and 110-nt duplication variants (marked with an orange bars). Guangzhou hMPV sequences are also highlighted by red bars in (**b**,**c**). (**d**) Heatmap of nucleotide identity matrix of hMPV strains. G genes are grouped by genetic lineages and arranged according to the isolation time.

**Figure 3:**
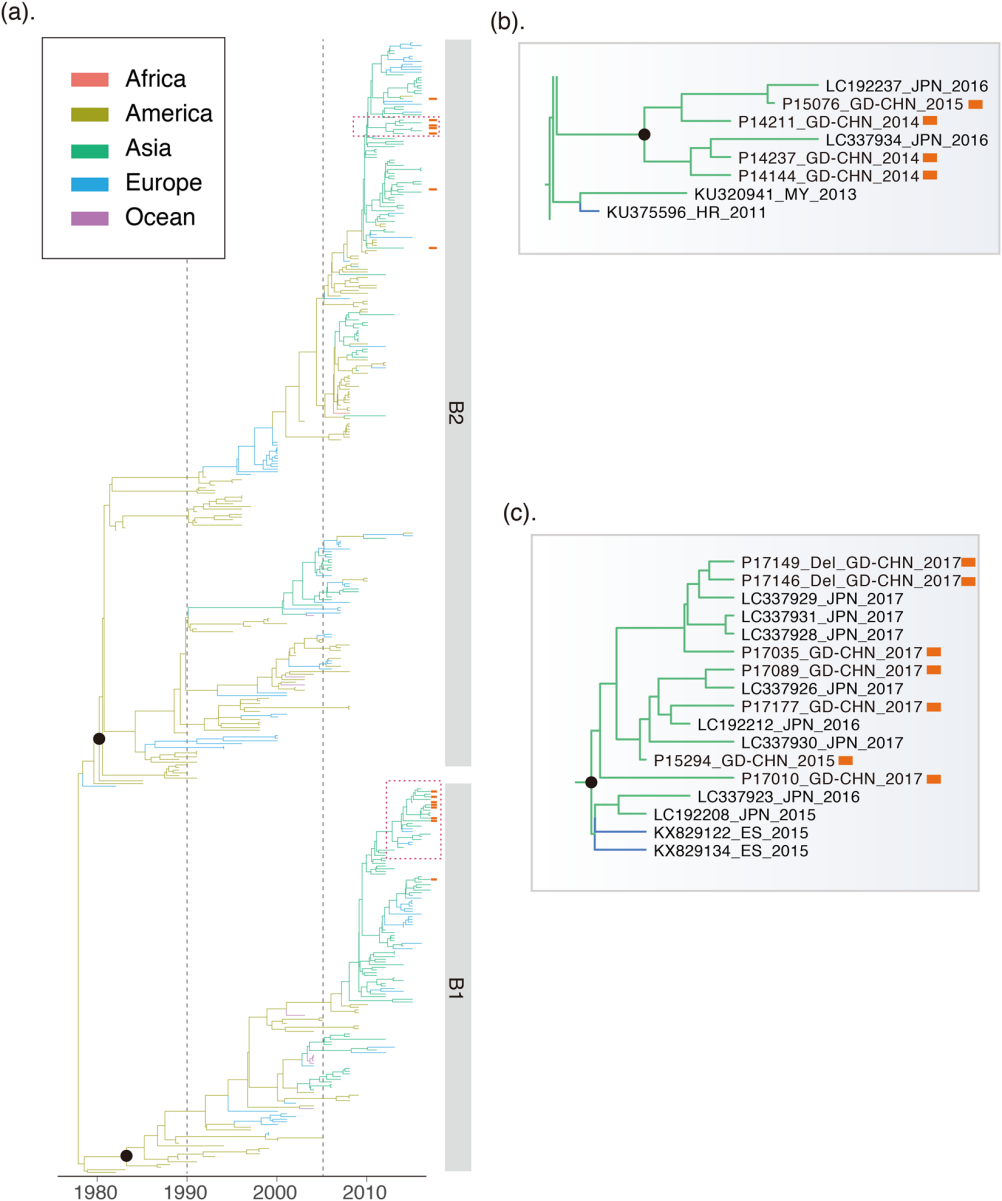
Genetic evolution and spatial spread of the genogroup B hMPV lineages. (**a**) Bayesian Maximum Clade Credibility (MCC) tree of G gene from genogroup B hMPV viruses. sequences reported in this study from Guangzhou are highlighted by red bars. Branch colours represent the most probable ancestral locations of each branch. Four major lineages of hMPV are observed, denoted B1 and B2. Black circles indicate posterior support >0.95 at the root node of each lineage. The clusters containing major circulating strains in Guangzhou are marked with box. These clusters are enlarged and names of strains are included in (**b**,**c**).

**Figure 4:**
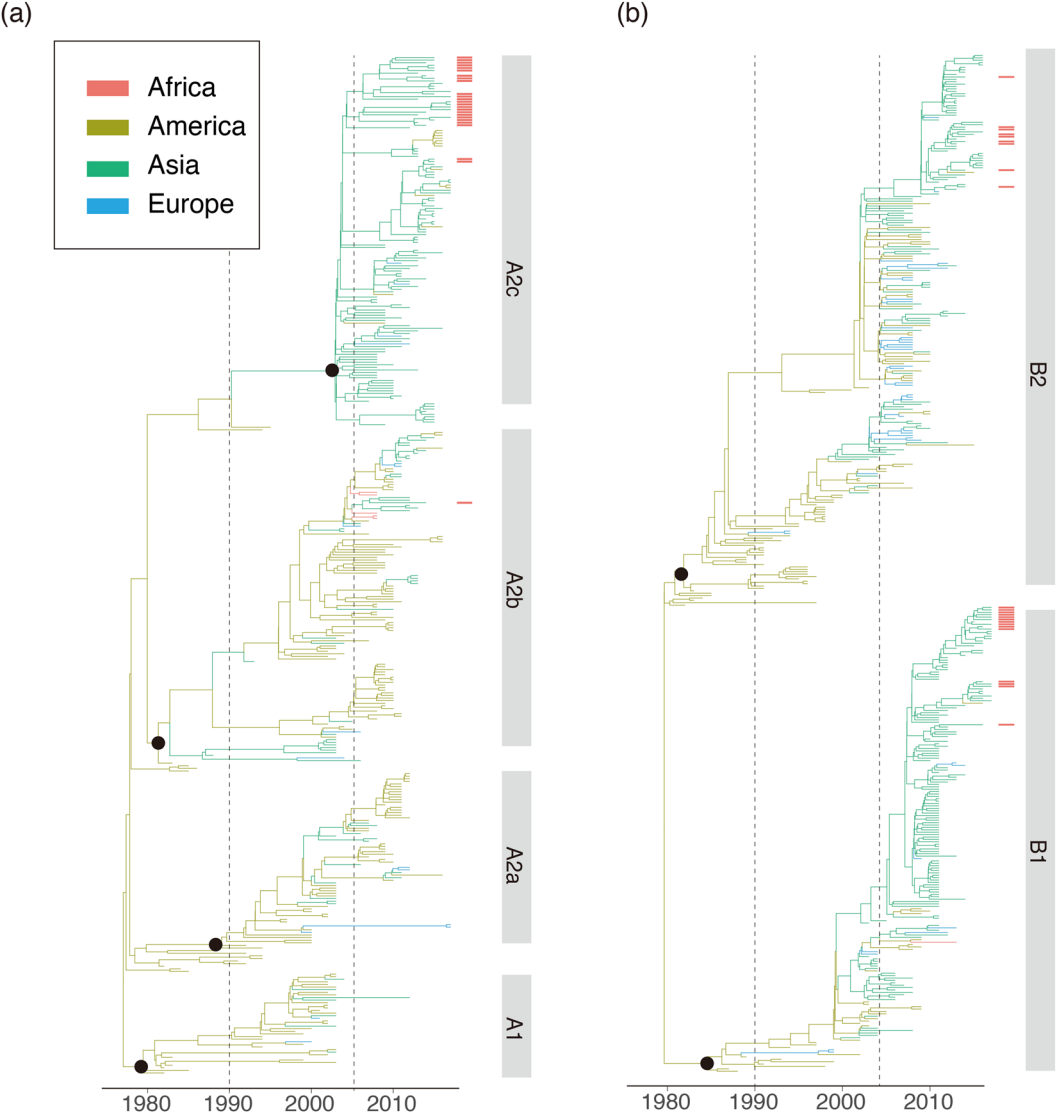
Bayesian Maximum Clade Credibility (MCC) tree of F gene from genogroup A (**a**) and genogroup B (**b**) hMPV viruses. sequences reported in this study from Guangzhou are highlighted by red bars. The color scheme of the phylogeny was the same as Figs 2 and 3.

To understand the lineage distribution and the cause(s) for the increase of the hMPV epidemic in 2017, 32 G genes and 51 F genes from 2013 through 2017 were sequenced. The evolution of hMPV at the global level was investigated by integrating all other publicly available G gene and F gene sequences (Materials and Methods). Due to the great genetic distance observed between two genogroups of hMPV (nucleotide identity of G gene between A and B groups is less than 57%), sequences from A and B genogroups were analyzed separately (Figs 2–4).

The hMPV A genogroup can be divided into four lineages (A1, A2a, A2b, A2c) based on the phylogeny topology (Figs 2a and 4a) and genetic diversity (Fig. 2d). The heat map of identity matrix clearly showed A1 strains were divergent from all A2 lineages (identity less than 85.2%), but similar genetic distances or identities were observed among A2a, A2b, and A2c (Fig. 2d, Table 1). Therefore, A2c was named as a novel A2b lineage in a previous study^23^ but marked as a novel lineage in this study due to the comparable genetic identities between each pair of the A2 sub-lineages (Fig. 2d, Table 1). For instances, the pairwise nucleotide identities between strains from A2b and A2c lineages were range from 87.5–93.3% which was similar with that observed between strains from A2a and A2b (86.0–93.3%). Within lineage, the divergent patterns showed by the G genes existed according to the time, seemingly having little correlation with the isolation date (Fig. 2d).

**Table 1:** Pairwise identities (%) of G genes from strains within and between hMPV lineages at both the nucleotide and amino acid levels (in bracket).

Lineages	A1	A2a	A2b	A2c
A1	89.9–100 (81.8–100)	77.6–85.2 (61.2–72.9)	77.7–85.0 (59.3–72.4)	77.9–84.3 (59.8–72.9)
A2a	—	91.9–100 (86.4–100)	86.0–93.3 (75.7–87.4)	86.1–92.8 (75.7–86.9)
A2b	—	—	91.1–100 (83.2–100)	87.5–93.3 (76.2–87.4)
A2c	—	—	—	91.1–100 (83.2–100)

### Global distribution of hMPV lineages

The phylogeographic analysis of the G gene showed a spatial pattern of hMPV lineages. Strains near the root of genogroup A primarily circulated in America (Fig. 2a). Although an American origin for the genogroup A of hMPV seems the most probable (posterior probability = 1.0), this might also be caused by sampling biases. Between 1990 and 2005, hMPV infections began to be detected in other continents. Notably, regional clusters of hMPV were observed in lineages A1 and A2a (Fig. 2a), suggesting the local circulation of these hMPV viruses. However, these circulations were always temporal as the regional phylogenetic clusters in A1 and A2a lineages were only persistent during 2–3 years (Fig. 2a).

**Figure 5:**
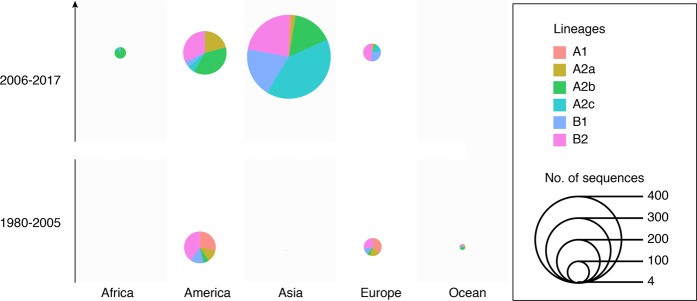
Continental distribution of hMPV genetic lineages in year 1980–2005 and 2006–2017. The number of lineages strains and spatial distribution are calculated according to the phylogeny of G genes in Figs 2 and 3.

In contrast, a cluster in A2b, early detected in America, dominantly circulated in Asian countries after 2005 when the hMPV surveillances started in these regions^22,36,37^. This cluster was persistent in Asia for more than 10 years, and the transmissions of this cluster of strains from Asia to other continents were observed. More obviously, the young lineage A2c most likely originated around 2001 (1997–2003, 95%HPD) and was well established in Asian countries. Current surveillance data showed A2c was now the major epidemic lineage in genogroup A. A similar pattern was observed for the F gene (Fig. 4). The A2c lineage of hMPV was endemic in Asia as the F gene phylogeny and introductions of A2c strains from Asia into other continents were observed after 2010 (Fig. 4a). In genogroup B, the sequences near the root of the phylogenetic tree were also mainly from America (Figs 3a and 4b). Lineages B1 and B2 of hMPV were established in Asian countries after 2010, and thereafter hMPV transmissions between Asia and Europe were frequently observed (Fig. 5).

### Local transmission of hMPV

The continental-specific distribution was observed for hMPV genetic lineages (Fig. 5). Then, the next intriguing question became how hMPV circulated at a country level. In particular, the increasing epidemic of hMPV in Guangzhou in 2017 was caused by the local strains circulating in previous seasons or the strains being imported from another region. The phylogenetic analyses of hMPV strains isolated in Guangzhou 2013–2017 showed the predominant lineage was varied in different years (Figs 2a and 3a). Based on the phylogeny of F genes, 26, 1, 15, and 10 strains were classified into A2c, A2b, B1, and B2 lineages, respectively (Fig. 4). In 2017, we identified one HMPV A2c strain with 111nt-dup in the G gene (P17072_GD-CHN_110ntDup_2017, Fig. 2b). This strain is closely related with the 111nt-dup hMPV novel variant most recently identified in Japan in 2017^24^. Notably, all these novel variants were scattered in a cluster of A2c lineage, indicating these variants were likely the result of multiple incidences of mutations rather than having evolved from a single mutation strain (Fig. 2a,b). In this scenario, the mutation leading to a duplication fragment in the G gene may easily have occurred for hMPV. In Guangzhou, hMPV infections were mainly caused by A2c, B1, and B2 lineage strains and none of the A1 and A2a lineage strains were identified in hMPV infection cases (Figs 2 and 4). Notably, in 2017, B1 lineage strains replaced the A2c and were likely the major causative strains leading to the increasing epidemiology of hMPV infections in Guangzhou (Figs 1, 3 and 4b). To trace the transmission of hMPV, we reconstructed the potential origins of Guangdong hMPV strains based on the phylogeny of the G gene. The hMPV strains isolated in Guangzhou in different years were always closely related (Figs 2b,c and 3b,c). However, the contemporary strains from Japan were always clustered together with strains of Guangzhou, highlighting the frequent transmissions among cities in China and Japan. The B1 strains causing the increasing hMPV epidemic in Guangzhou 2017 were more likely imported from Japan since closely related sequences were not found in Guangzhou but in Japan in the previous season (Fig. 3c).

## Discussion

Despite the first isolation in 2001, serological data suggested hMPV has been circulating globally for more than 50 years^1^. The great genetic distance between two genogroups (A and B) of hMPV indicates the origin of hMPV should be much earlier. To date, the molecular evolution, spatial distribution, and transmission of hMPV have not been adequately studied. Molecular surveillance will also be helpful to understand the transmission pathways and to confirm the source of infection, when it is analyzed in conjunction with standard epidemiological investigation. In this study, we performed molecular surveillance on hMPV in Guangzhou, China in 2013–2017. By integrating all publicly available data, we described the spatial dynamics of different hMPV lineages. The establishments of hMPV were identified in Asian countries after 2005. Moreover, frequent transmissions of hMPV strains and the seeding of novel variants were observed in China and Japan, highlighting the risk of an hMPV epidemic in these regions.

The spatial transmission pattern of a virus is very important for epidemic control and potential vaccine design. Viruses like H3N2 seasonal influenza virus are highly transmissible, and a new adaptive variant can rapidly spread globally after its emergence^38,39^. Thus, in the phylogeny of H3N2, it is rare to have a genetic cluster that is persistent and limited in a specific region. A sink-source model of H3N2 viral ecology is suggested in which new variants are seeded from the tropics (mainly East and Southeast Asia) to sink populations in temperate regions^39,40^. In contrast, viruses like enterovirus A 71 (EVA71) are more endemic, and different genetic subtypes circulate and persist in different regions. For instance, the C4a sub-genotype of EVA71 mainly circulates in mainland China, while the C5 genotype of EVA71 is mainly detected in Vietnam^41^. The establishment and prevalence of the specific sub-genotype of a viral species could be determined by environment factors, population immunity, and/or the genetic background of hosts in this region. Here, we trace the global as well as local hMPV distribution and transmission by integrating publicly available sequences and newly generated sequences. We find that hMPV was endemic in America before 1990, indicating the possible origin of currently circulating hMPV lineages. Notably, this could be the case because hMPV was limited to circulating in America or could be caused by sampling bias as there were few samplings on other continents before 1990 (Fig. 5). Further retrospective study of hMPV in other continents would improve our understanding of the origin of hMPV. Between 1990 and 2000, transmissions from America to other continents were observed. However, most of these introductions were transient since the local phylogenetic clusters of hMPV were not sustained in the phylogeny, indicating hMPV was still not well established in the regions. After 2005, clusters of hMPV mainly composed by strains in A2b and A2c lineages became dominant in Asian countries. Thereafter, transmissions from Asia to other continents were frequently identified through the phylogenetic trees (Figs 2a and 4a). These results suggest the establishment of A2b and A2c lineages in Asia after 2005, and more Asian countries became epicenters for hMPV. In this scenario, it is reasonable to have novel variants of hMPV first identified in Asian regions. The 180-nt and 111-nt duplicates of hMPV in the G gene were first reported in Japan in 2016 and 2017, and these newly emerged variants caused an increasing epidemic in Japan^23,24^. In Guangzhou, the duplicate variant was also detected and closely related with the strains identified in Japan with nucleotide identity at 99.3% (Fig. 2b). A2c was the predominant lineage identified in hMPV epidemics in Guangzhou, China 2013–2015. However, the increasing epidemic of hMPV infection in Guangzhou in 2017 was mainly caused by the B1 strains which were closely related viruses circulating in Japan in 2016 (Fig. 3). These results suggest the predominant hMPV lineage is varied among Asian countries in the same epidemic season, and rotation of these predominant lineages may be observed among these countries due to frequent viral transmissions.

Infections of hMPV were recently investigated in multiple regions of China^20–22,37,42^. Different seasonal patterns were observed in different climatic zones. In central China, hMPV is predominantly detected in winter and spring^20^. In northwest China, the annual peak of hMPV infection is observed in September through October^19^. However, in Guangzhou in southern China, the seasonal peaks of hMPV were detected mainly in March through May (Fig. 1). Although the temporal distribution and clinical symptoms of hMPV have been reported, the representative genetic sequences of hMPV in different regions of China are rarely investigated. Therefore, the geographic distributions and the transmission dynamics of hMPV lineages among different regions of China are still little understood. Our genetic description and analysis of hMPV viruses in southern China provide a baseline for future studies of the evolution and molecular epidemiology of this emerging virus in China.

In summary, our genetic and epidemiological analyses show that hMPV displayed different distribution and transmission patterns before 1990, 1990–2005, and 2006–2017. The firm establishment and continuous evolution of hMPV is observed in Asian countries after 2005, leading to the emergence of novel variants and the global transmission of these variants. In addition, close relations are observed among hMPV strains from China and Japan through the whole phylogenies. Therefore, careful monitoring of hMPV should be conducted throughout Asian countries to illustrate virus circulation in the area and sound an alarm for global hMPV surveillance.

## Supplementary information

Supplementary information
