# Supplementary material for: Epidemiology, evolution and transmission of human metapneumovirus in Guangzhou China, 2013–2017
Source: Sci Rep. 2019 Oct 1;9:14022. doi: 10.1038/s41598-019-50340-8 (PMC6773679; doi:10.1038/s41598-019-50340-8)
Supplement: Supplementary file 1 — Supplementary information [file 41598_2019_50340_MOESM1_ESM.docx]

Epidemiology, evolution and transmission of human metapneumovirus in Guangzhou China, 2013-2017

Running title: Epidemiology, evolution and transmission of hMPV

Lina Yi^1, 2*^, Lirong Zou^1*^, Jingju Peng^1, 3^, Jianxiang Yu^1^, Yingchao Song^1^, Lijun Liang^1^, Qianfang Guo^1^, Min Kang^1^, Changwen Ke^1^, Tie Song^1^, Jing Lu^1,2#^and Jie Wu^1#^

^1^Guangdong Provincial Center for Disease Control and Prevention, No. 160, Qunxian Road, Panyu District, Guangzhou, China.

^2^Guangdong Provincial Institute of Public Health, No. 160, Qunxian Road, Panyu District, Guangzhou, China.

^3^Southern Medical University, No. 1838, Shatai Road, Baiyun District, Guangzhou, People’s Republic of China

* These authors contributed equally to this work.

# These authors jointly supervised this work.

Correspondence to:

Prof. Jing Lu, Guangdong Provincial Institute of Public Health, No. 160, Qunxian Road, Panyu District, Guangzhou, China. Email: Jimlu0331@gmail.com

Prof. Jie Wu, Guangdong Provincial Center for Disease Control and Prevention, No.160, Qunxian Road, Panyu District, Guangzhou City, Guangdong Province, China. Email: wujie55@hotmail.com

Table S1. Accession number of public sequences used in this study

| G gene of hMPV Genogroup A (568) | | | | | | | |
| --- | --- | --- | --- | --- | --- | --- | --- |
| JF929831 | JF929832 | AY296014 | JF929833 | AY574224 | AF371337 | JX082173 | JX139006 |
| AY296015 | AY296016 | AY296017 | KU821121 | KF178956 | KC403977 | DQ362950 | EF571502 |
| KC562236 | JF929836 | JF929837 | JF929838 | AY574237 | KC562226 | JF929839 | JN184399 |
| KC403973 | KC403975 | KC403976 | AY574225 | AY574231 | AY574232 | AY574238 | AY574244 |
| AY296018 | AY485256 | AY485235 | KF178954 | KF178955 | DQ362948 | AY848886 | AY848897 |
| AY848899 | AY848901 | AY848882 | AY848883 | AY574228 | AY574226 | KF178958 | KF178959 |
| AY296019 | AY296020 | AY485254 | AY485236 | AY485255 | AY574246 | KF178953 | AY574243 |
| DQ362949 | AY848891 | AY485251 | AY485232 | AY485238 | AY485241 | AY485239 | AY848904 |
| AY848889 | AY848903 | KC403980 | AY848892 | AY848884 | AY848885 | AY848896 | AY848906 |
| AY848909 | AY848881 | AY848887 | AY848890 | AY848895 | AY848898 | AY848893 | AY848894 |
| KU320914 | JN974896 | LC192251 | LC360498 | MF045424 | LC192249 | LC270124 | KX829103 |
| LC275891 | LC192244 | KX829114 | KX829117 | KX829101 | LC192252 | KX829081 | LC192253 |
| KX829071 | KX829112 | LC192247 | LC192245 | LC192243 | MF462419 | LC192242 | KY474539 |
| LC192241 | LC192240 | LC192239 | LC192250 | LC192248 | KX829113 | LC192196 | KU320931 |
| LC192189 | LC192190 | LC192197 | KY474531 | KY474536 | KU320924 | KY474535 | KY474542 |
| KY474541 | KY474545 | KY474543 | KY474544 | LC192192 | LC192203 | KU176104 | KU176101 |
| KU176102 | KU176099 | LC192188 | KU320903 | LC192181 | LC192183 | KU320927 | LC192177 |
| JX082194 | JX139027 | JX082186 | JX139019 | JX082193 | JX139026 | JN974894 | JN974897 |
| KU375603 | JN831642 | JN974898 | KU320915 | KU320918 | LC316180 | KM361520 | JX082192 |
| JX139025 | KT191337 | LC192176 | LC192179 | KR138311 | KU320923 | JN200816 | LC192194 |
| LC192200 | LC192199 | LC192191 | LC192198 | LC192193 | LC192187 | KU320893 | KT191343 |
| KT191351 | KT191344 | KT191345 | JX082183 | JX139016 | JX082180 | JX139013 | JQ689395 |
| KU176103 | JX082175 | JX139008 | JX082188 | JX139021 | JX082187 | JX139020 | JX082189 |
| JX139022 | KU320904 | KU375594 | JX082176 | JX139009 | JX082179 | JX139012 | JQ689396 |
| JX082184 | JX139017 | KC731500 | JX082181 | JX139014 | JX082191 | JX139024 | JX082172 |
| JX139005 | JX082185 | JX139018 | KU320921 | KC731526 | KF178986 | KT191354 | JX082182 |
| JX139015 | JX082190 | JX139023 | JQ689393 | JQ689399 | JX082177 | JX139010 | LC192182 |
| LC192184 | LC192185 | GQ153651 | EF571504 | JN831639 | JN974900 | JN831640 | JN974893 |
| LC192202 | EF571505 | EF571506 | JQ689397 | JQ689394 | JQ689398 | JX082174 | JX139007 |
| HQ864259 | HQ864260 | HQ864233 | HQ864250 | EF571508 | JQ689390 | KC731524 | KU375605 |
| HQ599207 | JQ689391 | KU320892 | HQ599206 | KU320894 | KU375595 | KU320896 | KU320897 |
| KU320901 | LC192178 | KT191338 | KT191341 | KY474533 | KY474540 | LC192175 | KY474530 |
| KY474537 | KR138312 | KJ627377 | KC562243 | KJ627396 | KJ627381 | KJ627389 | KJ627411 |
| KU320920 | KU320910 | KU320899 | KU320900 | KF178975 | HQ599215 | KT191303 | KT032176 |
| KT032179 | KC403978 | KU320911 | KT191328 | KU320926 | KU320916 | KU320902 | KU320898 |
| KU320913 | KU320919 | KU320917 | KU320906 | KU320922 | KU320908 | KU320909 | KT191336 |
| KF178991 | KT191321 | KT191325 | KT191335 | KT191327 | KT191329 | KT191333 | HQ599216 |
| KC731502 | HQ599219 | LC192174 | KU375593 | KU375597 | KC795682 | KU320912 | HQ599214 |
| HQ599210 | KC795671 | KC795672 | HQ599213 | HQ599217 | HQ599218 | HQ599208 | HQ599209 |
| KF178989 | KF178995 | KU320905 | KF178996 | KF178998 | KF178988 | KT191349 | KU320895 |
| HQ599212 | KU320925 | KU320928 | KC731490 | KC731491 | KT191340 | KF178985 | KF178997 |
| KU320929 | KU320930 | KT191342 | KU320907 | KT191300 | KT191301 | KT191302 | KT191299 |
| KT191308 | KT191307 | KT191312 | KT191306 | KF178967 | KF178963 | KC731494 | KT032181 |
| HQ599211 | KT191334 | AY574234 | KF178962 | KF178973 | KT191309 | KC562224 | AY574241 |
| AY574227 | AY574240 | KF178971 | KF178972 | KF178964 | KC210062 | KC210060 | KC403983 |
| KC403984 | KJ627437 | KJ627406 | KJ627387 | KJ627399 | KJ627429 | KF178987 | KJ627426 |
| KJ627393 | KJ627410 | KJ627436 | KJ627403 | KJ627386 | KJ627404 | KJ627434 | KJ627424 |
| LC192170 | KC210064 | KC210069 | KC210075 | KC210061 | KJ627380 | KJ627408 | KJ627384 |
| KC210063 | KF178952 | KJ627405 | KJ627412 | KJ627422 | KJ627420 | KJ627430 | KF178984 |
| KF178992 | KF178994 | KF178983 | KJ627417 | KJ627385 | KJ627379 | KJ627402 | KF178974 |
| KJ627392 | KJ627382 | KJ627401 | KC210054 | KC210068 | KC210073 | KF178981 | KC210057 |
| KC210067 | KC210071 | KF178957 | KC562220 | KC562221 | EU259861 | EU259860 | EU259868 |
| HQ599204 | EU259869 | EU259872 | EU259863 | HQ599198 | HQ599202 | KC562240 | AY296030 |
| AY296031 | AY296029 | KT191320 | KT191332 | KT191346 | KT191353 | KT191339 | KT191350 |
| KT191347 | KT191348 | KT191352 | KT191331 | KT191310 | KT191313 | KT191315 | KT191322 |
| KT191316 | KT191314 | KT191317 | KT191319 | KT191318 | KT191323 | KT191330 | KT191324 |
| KC562225 | KC562233 | JF325878 | KC562241 | EU259870 | EU259862 | EU259859 | EU259874 |
| EU259867 | AY296023 | AY296024 | JF929842 | JN184400 | JF929843 | JF929844 | JF929847 |
| JF929841 | GQ888742 | JF929854 | JF929855 | JF929856 | JF929857 | AY574230 | AY574236 |
| AY574235 | AY485233 | AY485234 | JF929858 | AB503857 | AY848915 | DQ362952 | KC403979 |
| KC403982 | KF686742 | AY848914 | AY485250 | AY296027 | JF929859 | AY296021 | FJ168779 |
| MH150888 | MH150889 | AY848919 | AY297749 | AY485253 | AY848917 | AY848918 | AY848911 |
| AY848913 | AY848916 | AY848910 | AY848912 | AY296032 | AY296033 | AY574229 | GQ888740 |
| GQ888741 | JQ309681 | JQ689389 | KF178960 | KF178961 | KF178969 | KF178990 | KF178980 |
| KF178978 | KF178979 | KF178982 | KF178977 | KC403981 | AB846661 | KY474534 | KC210066 |
| KU375602 | KC210065 | KC210055 | KC210072 | KF178993 | KU375604 | KC210056 | KC210076 |
| KT032178 | KC210074 | KJ627398 | KJ627416 | KJ627425 | KJ627413 | KJ627423 | KJ627428 |
| KJ627427 | KJ627395 | KJ627433 | KJ627390 | KJ627378 | KJ627388 | KJ627394 | KJ627407 |
| KJ627415 | KJ627418 | KJ627421 | KJ627419 | KF178966 | JQ309676 | KF178965 | KF178970 |
| KF178968 | JQ309677 | KC210059 | JF929840 | JF929848 | AY296026 | AY296028 | AY296025 |
| JF929849 | JF929845 | JF929846 | JF929853 | JF929851 | JF929852 | AY296022 | JF929850 |
| G gene of hMPV Genogroup B (410) | | | | | | | |
| AB846660 | AY296034 | AY296035 | AY296036 | AY296037 | AY296038 | AY296039 | AY296040 |
| AY296041 | AY296042 | AY296043 | AY296044 | AY296045 | AY296046 | AY296047 | AY297748 |
| AY485242 | AY485243 | AY485244 | AY485245 | AY485246 | AY485247 | AY485248 | AY485249 |
| AY485252 | AY525843 | AY574247 | AY848859 | AY848860 | AY848861 | AY848862 | AY848863 |
| AY848864 | AY848865 | AY848866 | AY848867 | AY848868 | AY848869 | AY848870 | AY848875 |
| AY848877 | AY848878 | AY848879 | AY848880 | DQ312443 | DQ312453 | DQ312457 | DQ312460 |
| DQ362958 | DQ393715 | EF535506 | EF571500 | EF571501 | EF571503 | EF571507 | EF571509 |
| EF571510 | EF571511 | EU259877 | FJ168778 | GQ888739 | GQ888743 | HM197719 | HQ599199 |
| HQ599200 | HQ599201 | JF325881 | JF929860 | JF929861 | JF929862 | JF929863 | JF929865 |
| JF929868 | JF929870 | JF929871 | JF929874 | JF929875 | JF929876 | JF929877 | JF929878 |
| JF929879 | JF929880 | JF929881 | JF929882 | JF929884 | JF929886 | JF929887 | JF929888 |
| JF929889 | JF929890 | JF929891 | JF929892 | JF929893 | JF929894 | JF929895 | JF929896 |
| JF929897 | JF929898 | JF929899 | JF929901 | JF929902 | JF929903 | JN184401 | JN184402 |
| JN831630 | JN831631 | JN831632 | JN831633 | JN831634 | JN831636 | JN831637 | JN831641 |
| JQ309673 | JQ309675 | JQ309678 | JQ309679 | JQ309680 | JQ309682 | JQ513461 | JQ513462 |
| JQ513464 | JQ513465 | JQ513466 | JQ513467 | JQ513468 | JQ513469 | JQ513470 | JQ513471 |
| JQ513472 | JQ513473 | JQ513475 | JQ513477 | JQ513478 | JQ513479 | JQ513480 | JQ513481 |
| JQ513482 | JQ513484 | JQ689392 | JX082178 | JX139011 | JX997977 | KC210077 | KC210078 |
| KC210079 | KC210080 | KC210081 | KC210082 | KC403971 | KC403972 | KC403974 | KC470020 |
| KC470021 | KC562219 | KC562222 | KC562223 | KC562227 | KC562228 | KC562229 | KC562230 |
| KC562231 | KC562232 | KC562234 | KC562235 | KC562237 | KC562238 | KC562239 | KC562242 |
| KC562244 | KC731486 | KC731487 | KC731489 | KC731492 | KC731497 | KC731498 | KC731505 |
| KC731506 | KC731509 | KC731511 | KC731513 | KC731518 | KC731521 | KC731523 | KC795615 |
| KC795616 | KC795617 | KC795619 | KC795620 | KC795621 | KC795622 | KC795626 | KC795662 |
| KC795663 | KC795665 | KC795666 | KC795670 | KF178999 | KF179000 | KF179001 | KF179002 |
| KF179003 | KF179004 | KF179005 | KF179006 | KF179007 | KF179008 | KF179009 | KF179010 |
| KF179011 | KF179012 | KF179013 | KF179014 | KF179015 | KF179016 | KF179017 | KF179018 |
| KF179019 | KF179020 | KF179021 | KF179022 | KF179023 | KF179024 | KF179025 | KF179026 |
| KF179027 | KF179028 | KF179029 | KF179030 | KF179031 | KF179032 | KF179033 | KF179034 |
| KF179035 | KF179036 | KF179037 | KF179038 | KF179039 | KF179040 | KF179041 | KF179042 |
| KF179043 | KF179044 | KF179045 | KF516922 | KF530155 | KF530159 | KF530163 | KF530164 |
| KF530167 | KF530171 | KF530172 | KF530173 | KF530174 | KF530176 | KF530178 | KF530179 |
| KJ627383 | KJ627391 | KJ627397 | KJ627400 | KJ627414 | KJ627431 | KJ627432 | KJ627435 |
| KT032173 | KT032174 | KT032175 | KT032177 | KT032180 | KU176100 | KU176106 | KU320932 |
| KU320933 | KU320934 | KU320935 | KU320936 | KU320937 | KU320938 | KU320939 | KU320940 |
| KU320941 | KU320942 | KU320943 | KU320944 | KU320945 | KU320946 | KU320947 | KU320948 |
| KU320949 | KU320950 | KU320951 | KU320952 | KU320953 | KU320954 | KU320955 | KU320956 |
| KU320957 | KU320958 | KU320959 | KU320960 | KU320961 | KU320962 | KU320963 | KU320964 |
| KU320965 | KU320966 | KU320967 | KU320968 | KU320969 | KU320970 | KU320971 | KU320972 |
| KU320973 | KU375596 | KU375598 | KU375599 | KU375600 | KU375601 | KU375606 | KU375607 |
| KU375608 | KX829120 | KX829121 | KX829122 | KX829123 | KX829124 | KX829125 | KX829126 |
| KX829127 | KX829128 | KX829131 | KX829132 | KX829133 | KX829134 | KX829135 | KX829136 |
| KX829137 | KX829138 | KX829143 | KX829144 | KX829145 | KX829147 | KX829148 | KX829150 |
| KX829151 | KX829152 | KX829154 | KX829155 | KX829156 | KX829157 | KX829158 | KX829161 |
| KX829163 | KX829166 | KX829167 | KX829168 | KY967366 | LC192204 | LC192205 | LC192206 |
| LC192207 | LC192208 | LC192209 | LC192210 | LC192211 | LC192212 | LC192213 | LC192214 |
| LC192215 | LC192216 | LC192217 | LC192219 | LC192220 | LC192221 | LC192222 | LC192223 |
| LC192224 | LC192228 | LC192229 | LC192230 | LC192231 | LC192233 | LC192234 | LC192235 |
| LC192236 | LC192237 | LC192238 | LC337921 | LC337922 | LC337923 | LC337924 | LC337925 |
| LC337926 | LC337927 | LC337928 | LC337929 | LC337930 | LC337931 | LC337932 | LC337933 |
| LC337934 | LC337935 | LC337936 | LC337937 | LC337938 | LC337939 | LC337940 | LC337941 |
| MF045425 | MG431250 |  |  |  |  |  |  |
| F gene of hMPV Genogroup A (365) | | | | | | | |
| AB503857 | AB618741 | AB618743 | AB618747 | AB693958 | AB846659 | AF371337 | AY145294 |
| AY145296 | AY145297 | AY145299 | AY145301 | AY297749 | AY304360 | AY530090 | AY530091 |
| AY530092 | AY530093 | AY530095 | AY622381 | DQ061252 | DQ362937 | DQ362939 | DQ362940 |
| DQ362941 | DQ841214 | EF397621 | EF397627 | EF589610 | EU857542 | EU857543 | EU857544 |
| EU857545 | EU857546 | EU857547 | EU857549 | EU857550 | EU857551 | EU857552 | EU857553 |
| EU857554 | EU857555 | EU857556 | EU857557 | EU857558 | EU857559 | EU857560 | EU857561 |
| EU857562 | EU857563 | EU857564 | EU857565 | EU857566 | EU857608 | EU857609 | EU857610 |
| FJ168779 | GQ153651 | GU048721 | GU048735 | GU048738 | GU048739 | GU048742 | GU048744 |
| GU048745 | HQ262552 | HQ262553 | HQ262554 | HQ262556 | HQ262560 | HQ262562 | HQ262563 |
| HQ262565 | HQ262567 | HQ262568 | HQ864307 | HQ864309 | HQ864312 | JF325877 | JN184399 |
| JN184400 | JN831625 | JN831628 | JN974887 | JQ413388 | JQ413389 | JQ413391 | JQ413392 |
| JQ413393 | JQ413394 | JQ413395 | JQ413396 | JQ413405 | JQ413406 | JQ413407 | JQ413408 |
| JQ413409 | JQ413410 | JQ413411 | JQ413412 | JQ745052 | JQ745068 | JQ888113 | JQ888114 |
| JQ888115 | JQ888116 | JQ888118 | KC403973 | KC403975 | KC403976 | KC403977 | KC403978 |
| KC403979 | KC403980 | KC403981 | KC403982 | KC403983 | KC403984 | KC562220 | KC562221 |
| KC562224 | KC562225 | KC562226 | KC562233 | KC562236 | KC562240 | KC562241 | KC562243 |
| KC562245 | KC588902 | KC588905 | KC588906 | KC731508 | KC731510 | KC731514 | KC731515 |
| KC731525 | KC731526 | KC731527 | KF192711 | KF192712 | KF192713 | KF192714 | KF192715 |
| KF192716 | KF192717 | KF192718 | KF192719 | KF192720 | KF192721 | KF192722 | KF192723 |
| KF192724 | KF192725 | KF192726 | KF192727 | KF192728 | KF192729 | KF192730 | KF192731 |
| KF192732 | KF192733 | KF192734 | KF192735 | KF192736 | KF192737 | KF192738 | KF192739 |
| KF192740 | KF192741 | KF192742 | KF192743 | KF192744 | KF192745 | KF192746 | KF192747 |
| KF192748 | KF192749 | KF192750 | KF192751 | KF192752 | KF192753 | KF192754 | KF192755 |
| KF192756 | KF192757 | KF192758 | KF192759 | KF192760 | KF192761 | KF192762 | KF192763 |
| KF686742 | KJ196300 | KJ196301 | KJ196302 | KJ196303 | KJ196304 | KJ196305 | KJ196306 |
| KJ196307 | KJ196309 | KJ196310 | KJ196311 | KJ196312 | KJ196314 | KJ627377 | KJ627378 |
| KJ627379 | KJ627380 | KJ627381 | KJ627382 | KJ627384 | KJ627385 | KJ627386 | KJ627387 |
| KJ627388 | KJ627389 | KJ627390 | KJ627392 | KJ627393 | KJ627394 | KJ627395 | KJ627396 |
| KJ627398 | KJ627399 | KJ627401 | KJ627402 | KJ627403 | KJ627404 | KJ627405 | KJ627406 |
| KJ627407 | KJ627408 | KJ627410 | KJ627411 | KJ627412 | KJ627413 | KJ627415 | KJ627416 |
| KJ627417 | KJ627418 | KJ627419 | KJ627420 | KJ627421 | KJ627422 | KJ627423 | KJ627424 |
| KJ627425 | KJ627426 | KJ627427 | KJ627428 | KJ627429 | KJ627430 | KJ627433 | KJ627434 |
| KJ627436 | KJ627437 | KM361520 | KM408076 | KU254673 | KU254680 | KU254685 | KU254687 |
| KU254689 | KU254694 | KU254695 | KU254702 | KU254703 | KU254704 | KU254706 | KU254707 |
| KU254708 | KU254709 | KU254717 | KU254719 | KU254720 | KU254724 | KU254730 | KU254731 |
| KU254736 | KU254747 | KU254751 | KU254755 | KU375609 | KU375610 | KU375611 | KU375617 |
| KU375618 | KU375622 | KU375623 | KU375624 | KU821121 | KY474529 | KY474530 | KY474532 |
| KY474533 | KY474534 | KY474535 | KY474536 | KY474537 | KY474538 | KY474539 | KY474540 |
| KY474541 | KY474542 | KY474543 | KY474544 | KY474545 | LC337669 | LC337670 | LC337671 |
| LC337672 | LC337673 | LC337674 | LC337675 | LC337676 | LC337677 | LC337678 | LC337679 |
| LC337680 | LC337681 | LC337682 | LC337683 | LC337684 | LC337685 | LC337686 | LC337687 |
| LC337688 | LC337689 | LC337690 | LC337691 | LC337692 | LC337693 | LC337694 | LC337695 |
| LC337696 | LC337697 | LC337698 | LC337699 | LC337700 | LC337701 | LC337756 | LC337757 |
| LC337758 | LC337759 | LC337760 | LC337761 | LC337762 | LC337763 | LC337764 | LC337765 |
| LC337766 | LC337767 | LC337768 | LC337769 | LC337770 | LC337771 | LC337772 | LC360499 |
| MF045424 | MF159108 | MF166862 | MH150888 | MH150889 |  |  |  |
| F gene of hMPV Genogroup B (402) | | | | | | | |
| AB618735 | AB618737 | AB618739 | AB618749 | AB618756 | AB618758 | AB618760 | AB618764 |
| AB693954 | AB693956 | AB846658 | AY145287 | AY145289 | AY145290 | AY145291 | AY145292 |
| AY145293 | AY145295 | AY145298 | AY145300 | AY297748 | AY304361 | AY304362 | AY525843 |
| AY530089 | AY530094 | DQ061253 | DQ061254 | DQ362943 | DQ843658 | EF081369 | EF535506 |
| EU857567 | EU857569 | EU857570 | EU857572 | EU857573 | EU857574 | EU857575 | EU857576 |
| EU857577 | EU857579 | EU857580 | EU857581 | EU857582 | EU857583 | EU857584 | EU857586 |
| EU857587 | EU857588 | EU857589 | EU857591 | EU857593 | EU857594 | EU857595 | EU857596 |
| EU857597 | EU857598 | EU857599 | EU857600 | EU857601 | EU857602 | EU857606 | EU857607 |
| FJ168778 | GU048708 | GU048710 | GU048711 | GU048712 | GU048713 | GU048714 | GU048715 |
| GU048716 | GU048717 | GU048719 | GU048720 | GU048722 | GU048723 | GU048724 | GU048725 |
| GU048727 | GU048729 | GU048730 | GU048731 | GU048732 | GU048733 | GU048734 | GU048736 |
| GU048737 | GU048740 | GU048741 | GU048743 | HM197719 | HQ262551 | HQ262555 | HQ262557 |
| HQ262559 | HQ262564 | HQ262566 | HQ864261 | HQ864262 | HQ864263 | HQ864264 | HQ864265 |
| HQ864266 | HQ864268 | HQ864273 | HQ864274 | HQ864276 | HQ864278 | HQ864279 | HQ864281 |
| HQ864282 | HQ864283 | HQ864284 | HQ864285 | HQ864287 | HQ864289 | HQ864290 | HQ864294 |
| HQ864297 | HQ864298 | HQ864301 | HQ864302 | JF325880 | JN184401 | JN184402 | JN831611 |
| JN831613 | JN831614 | JN831615 | JN831616 | JN831617 | JN831618 | JN831619 | JN831620 |
| JN831621 | JN831622 | JN831623 | JN831624 | JN831627 | JN974890 | JQ181560 | JQ181561 |
| JQ181562 | JQ181563 | JQ181564 | JQ181565 | JQ181566 | JQ181567 | JQ181569 | JQ181570 |
| JQ181571 | JQ181572 | JQ181573 | JQ181574 | JQ181575 | JQ181576 | JQ181577 | JQ181578 |
| JQ181580 | JQ181581 | JQ181583 | JQ181584 | JQ413397 | JQ413398 | JQ413399 | JQ413400 |
| JQ413401 | JQ413402 | JQ413403 | JQ745049 | JQ745050 | JQ745051 | JQ745053 | JQ745054 |
| JQ745055 | JQ745056 | JQ745058 | JQ745059 | JQ745060 | JQ745061 | JQ745063 | JQ745064 |
| JQ745065 | JQ745066 | JQ745067 | JQ745069 | JQ745070 | JQ745071 | JQ745072 | JQ745073 |
| JQ745074 | JQ745075 | JQ745076 | JQ745077 | JQ745078 | JQ745079 | JQ745080 | JQ745081 |
| JQ745082 | JQ745084 | JQ745085 | JQ745086 | JQ745087 | JQ745088 | JQ745089 | JQ745090 |
| JQ745091 | JQ745092 | JQ745093 | JQ745094 | KC403971 | KC403972 | KC403974 | KC470017 |
| KC470018 | KC470019 | KC562219 | KC562222 | KC562223 | KC562227 | KC562228 | KC562229 |
| KC562230 | KC562231 | KC562232 | KC562234 | KC562235 | KC562237 | KC562238 | KC562239 |
| KC562242 | KC562244 | KC588903 | KC588904 | KC731509 | KC731511 | KC731518 | KC731521 |
| KC731522 | KC731523 | KF192764 | KF192765 | KF192766 | KF192767 | KF192768 | KF192769 |
| KF192770 | KF192771 | KF192773 | KF192774 | KF192775 | KF192776 | KF192777 | KF192778 |
| KF192779 | KF192780 | KF192781 | KF192782 | KF192783 | KF192784 | KF192785 | KF192786 |
| KF192787 | KF192788 | KF192789 | KF192790 | KF192791 | KF192792 | KF192793 | KF192794 |
| KF192795 | KF192796 | KF192797 | KF192798 | KF192799 | KF192800 | KF192801 | KF192802 |
| KF192803 | KF516922 | KF530155 | KF530163 | KF530164 | KF530167 | KF530171 | KF530173 |
| KF530176 | KF530178 | KF530179 | KJ196315 | KJ196316 | KJ196317 | KJ196318 | KJ627383 |
| KJ627391 | KJ627397 | KJ627400 | KJ627414 | KJ627431 | KJ627432 | KJ627435 | KU254674 |
| KU254675 | KU254676 | KU254677 | KU254678 | KU254679 | KU254681 | KU254682 | KU254684 |
| KU254688 | KU254691 | KU254692 | KU254693 | KU254697 | KU254698 | KU254699 | KU254705 |
| KU254716 | KU254718 | KU254721 | KU254725 | KU254727 | KU254728 | KU254734 | KU254737 |
| KU254741 | KU254742 | KU254743 | KU254744 | KU254746 | KU254756 | KU254757 | KU375612 |
| KU375613 | KU375614 | KU375615 | KU375616 | KU375619 | KU375620 | KU375621 | KY967366 |
| LC337702 | LC337703 | LC337704 | LC337705 | LC337706 | LC337707 | LC337708 | LC337709 |
| LC337710 | LC337711 | LC337712 | LC337713 | LC337714 | LC337715 | LC337716 | LC337717 |
| LC337718 | LC337719 | LC337720 | LC337721 | LC337722 | LC337723 | LC337724 | LC337725 |
| LC337726 | LC337727 | LC337728 | LC337729 | LC337730 | LC337731 | LC337732 | LC337733 |
| LC337734 | LC337735 | LC337736 | LC337737 | LC337738 | LC337739 | LC337740 | LC337741 |
| LC337742 | LC337743 | LC337744 | LC337745 | LC337746 | LC337747 | LC337748 | LC337749 |
| LC337750 | LC337751 | LC337752 | LC337753 | LC337754 | LC337755 | MF045425 | MG431250 |
| MG773272 | MH685676 |  |  |  |  |  |  |
